# NAD^+^ metabolism-based immunoregulation and therapeutic potential

**DOI:** 10.1186/s13578-023-01031-5

**Published:** 2023-05-10

**Authors:** Jiankai Fang, Wangwang Chen, Pengbo Hou, Zhanhong Liu, Muqiu Zuo, Shisong Liu, Chao Feng, Yuyi Han, Peishan Li, Yufang Shi, Changshun Shao

**Affiliations:** 1 Institutes for Translational Medicine, State Key Laboratory of Radiation Medicine and Protection, The First Affiliated Hospital of Soochow University, Suzhou Medical College of Soochow University, Suzhou, Jiangsu China; 2 Laboratory Animal Center, Suzhou Medical College of Soochow University, Suzhou, Jiangsu China; 3 Department of Experimental Medicine and Biochemical Sciences, TOR, University of Rome Tor Vergata, Rome, Italy; 4 Shanghai Institute of Nutrition and Health, Shanghai Institutes for Biological Sciences, Chinese Academy of Sciences, Shanghai, China

**Keywords:** Nicotinamide adenine dinucleotide (NAD^+^), Immunoregulation, Metabolic homeostasis, Plasticity, Disease therapy

## Abstract

Nicotinamide adenine dinucleotide (NAD^+^) is a critical metabolite that acts as a cofactor in energy metabolism, and serves as a cosubstrate for non-redox NAD^+^-dependent enzymes, including sirtuins, CD38 and poly(ADP-ribose) polymerases. NAD^+^ metabolism can regulate functionality attributes of innate and adaptive immune cells and contribute to inflammatory responses. Thus, the manipulation of NAD^+^ bioavailability can reshape the courses of immunological diseases. Here, we review the basics of NAD^+^ biochemistry and its roles in the immune response, and discuss current challenges and the future translational potential of NAD^+^ research in the development of therapeutics for inflammatory diseases, such as COVID-19.

## Key points

The dynamics of NAD^+^ homeostasis is characterized by a balance of synthesis, consumption and regeneration.

The role of NAD^+^ in immunomodulation is context and signaling pathway specific.

The manipulation of NAD^+^ bioavailability represents a promising therapeutic strategy for inflammatory diseases.

## Introduction

Nicotinamide adenine dinucleotide (NAD^+^), one of the most abundant molecules in the body, is required for over 500 different enzymatic reactions and participates in almost all known biological processes [1–3]. It was first described in 1906 by Harden and Young as a cell component that enhanced alcohol fermentation in yeast [4]. NAD^+^ research went through a period of relative dormancy until its resurrection 20 years ago. Much of the renewed interest in NAD^+^ was attributed to the sirtuins, a family of NAD^+^-dependent protein deacetylases. In this context, NAD^+^, as an essential cosubstrate for the activity of sirtuins, participates in the regulation of energy homeostasis, ageing and longevity [5–8].

## NAD^+^ homeostasis

### NAD^+^ and NADH

**Fig. 1:**
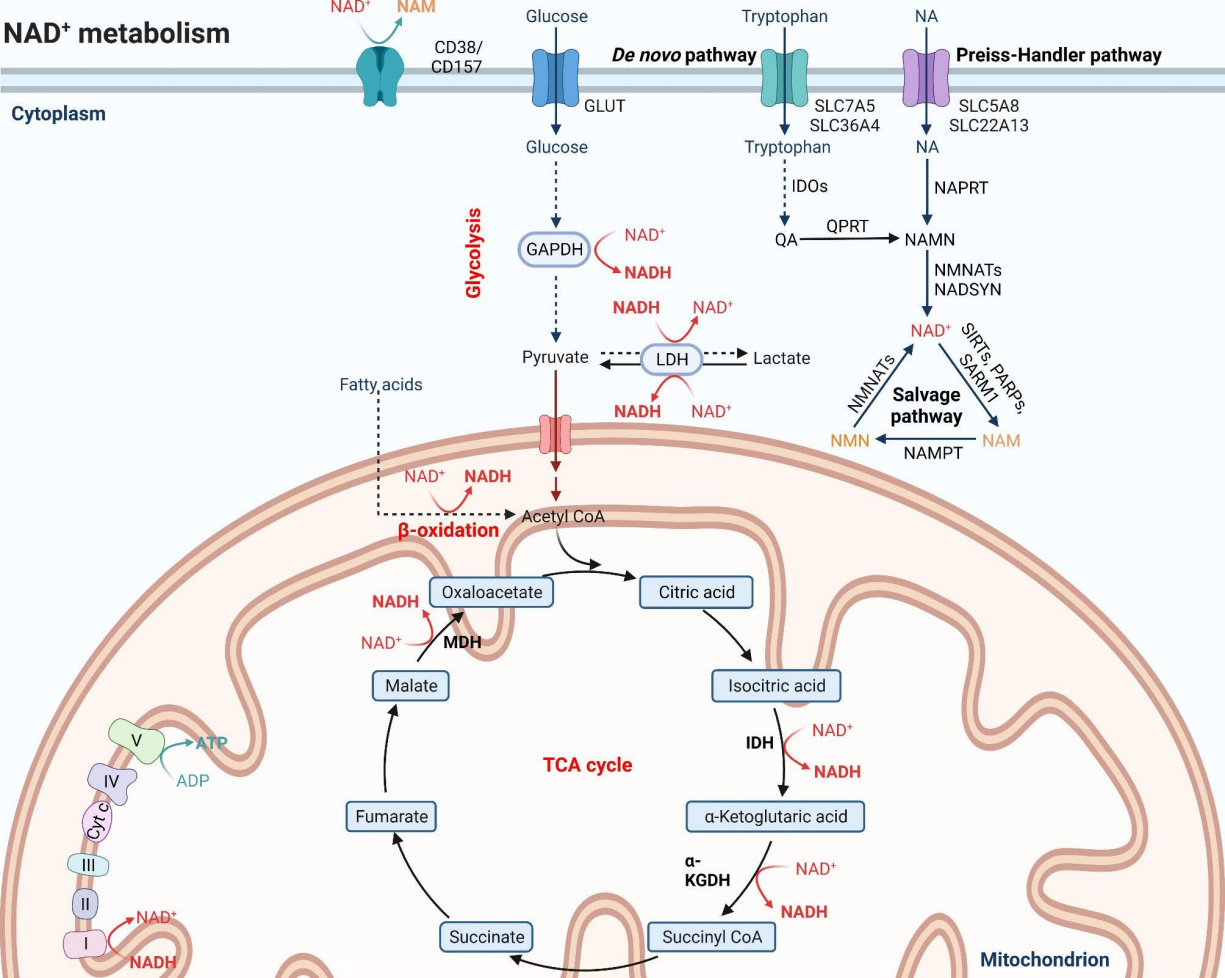
NAD^+^ metabolism. NAD^+^ levels are maintained by three independent biosynthetic pathways. The *de novo* synthesis pathway converts tryptophan to quinolinic acid (QA) via a series of enzymatic steps, in which indoleamine-2,3-dioxygenase (IDO) is a rate-limiting enzyme that catalyzes the first step and the conversion of QA to nicotinate mononucleotide (NAMN) is the ultimate bottleneck step catalyzed by quinolinate phosphoribosyltransferase (QPRT). The Preiss-Handler pathway uses dietary nicotinic acid (NA) to generate NAMN through nicotinate phosphoribosyltransferase (NAPRT). NAMN is converted to NAD^+^ by the sequential actions of nicotinamide mononucleotide adenylyl transferases (NMNATs) and NAD^+^ synthetase (NADSYN). The NAD^+^ salvage pathway recycles nicotinamide (NAM) generated as a by-product of the enzymatic activities of NAD^+^-consuming proteins (sirtuins, poly(ADP-ribose) polymerases (PARPs) and the NAD^+^ glycohydrolases CD38, CD157 and SARM1), into nicotinamide mononucleotide (NMN) via the rate-limiting enzyme NAM phosphoribosyltransferase (NAMPT). NMN is then converted into NAD^+^ via the different NMNATs. These renascent NAD^+^ receives a hydride to yield the reduced form NADH, thereby driving various metabolic processes including glycolysis, the tricarboxylic acid (TCA) cycle and β-oxidation of fatty acids. On the contrary, NADH provides an electron pair to drive oxidative phosphorylation (OXPHOS) for the generation of ATP in the mitochondria and the conversion of lactate to pyruvate in the cytoplasm, which are accompanied by intracellular NAD^+^ regeneration.

The ability of NAD^+^ to accept a hydride ion, forming its reduced form NADH, is instrumental for redox reactions in the cytosol and mitochondria and regulates the activity of dehydrogenases in multiple catabolic processes. Specifically, NAD^+^ serves as a cofactor for oxidoreductases involved in (1) glycolysis (glyceraldehyde phosphate dehydrogenase, GAPDH), (2) oxidative decarboxylation of pyruvate to acetyl-CoA (pyruvate dehydrogenase), (3) tricarboxylic acid cycle (α-ketoglutarate, isocitrate and malate dehydrogenases), (4) β-oxidation of fatty acid (3-hydroxyacyl-CoA dehydrogenase) and (5) alcohol metabolism (alcohol and aldehyde dehydrogenases), thereby harvesting energy in the form of ATP from fuel substrates, such as glucose, amino acids and fatty acids (Fig. 1). Overall, fuel cannot be metabolized and converted into energy without NAD^+^ [9, 10].

The ability of NADH to donate an electron, forming its oxidative version NAD^+^, is also essential for certain redox reactions. The reduction of pyruvate to lactate catalyzed by lactate dehydrogenase harvests electrons from NADH, thereby recycling cellular NAD^+^ to fuel ongoing glycolytic flows [11]. Likewise, NADH also acts as a cofactor for polyunsaturated fatty acid-related desaturases. This process is an alternative mechanism to regenerate cellular NAD^+^ to assist in glycolysis [12]. Finally, these high-energy electrons in NADH are transported to the mitochondrial electron transport chain (ETC) to drive oxidative phosphorylation (OXPHOS) under aerobic conditions.

### NADP^+^ and NADPH

Approximately 10% of cellular NAD^+^ can be phosphorylated at the adenosine riboside site to form NADP^+^ via NAD^+^ kinases [13], which acts as a hydride acceptor to form NADPH and is used in anabolic reactions and stress resistance [14, 15]. Like NAD^+^, NADP^+^ serves as a cofactor for the rate-limiting step of the pentose-phosphate pathway, which operates in parallel with glycolysis in the cytosol and is responsible for approximately 10–20% of glucose utilization [16]. In this process, diverse metabolic building blocks for the synthesis of nucleotides and aromatic amino acids are produced, which is simultaneously accompanied by substantial generation of cytosolic NADPH [17].

NADPH in turn acts as a coenzyme for reductive biosynthesis of fatty acids, cholesterol and steroids from acetyl-CoA [18], which is essential for alternative activation of macrophages during experimental helminth infection [19]. In addition, NADPH oxidases produce oxygen free radicals from NADPH during the respiratory burst of neutrophils to defend against invasive pathogens [20, 21]. Moreover, glutathione reductase uses NADPH to catalyze the reduction of glutathione disulfide to glutathione in response to oxidative stress [22]. Furthermore, a new identified hydride transfer complex (HTC) in the cytoplasm, assembled by malate dehydrogenase 1, malic enzyme 1 and cytosolic pyruvate carboxylase, transfers the hydride ion from NADH to NADP^+^, thereby regenerating NAD^+^, supplying NADPH for anabolic reactions and redox defenses, and hence conferring fitness to cancer cells under hypoxia or mitochondrial dysfunction [23, 24].

### NAD^+^ biosynthesis

Under normal physiological conditions, a balance between biosynthesis and consumption is sustained to maintain NAD^+^ homeostasis in mammalian cells. NAD^+^ can be produced from four dietary precursors, including nicotinamide (NAM), nicotinamide riboside (NR), nicotinamide mononucleotide (NMN) and nicotinic acid (NA), through different metabolic pathways. Alternatively, the *de novo* synthesis pathway can convert the essential amino acid tryptophan to NAD^+^ via eight sequential steps [25, 26]. The enzyme indole-2,3-dioxygenase 1 (IDO1) in the first committed step of the *de novo* synthesis pathway metabolizes tryptophan to the immunosuppressive metabolite kynurenine in the context of immune challenges [27–29]. The kynurenine is further metabolized through the subsequent *de novo* synthesis pathway to quinolinic acid (QA), which is converted by the enzyme quinolate phosphoribosyltransferase (QPRT) to NAD^+^ in immune cells such as macrophages [13, 30].

The Preiss-Handler pathway can convert dietary NA to NAD^+^ biosynthesis via three steps. The enzyme NA phosphoribosyltransferase (NAPRT) metabolizes NA to the intermediate NA mononucleotide (NAMN), which is then transformed into NA adenine dinucleotide (NAAD) by NMN adenylyltransferases (NMNATs). The final step catalyzed by NAD synthetase (NADS) commits the Preiss-Handler pathway to NAD^+^ production.

The salvage pathway contributes to most NAD^+^ production from system-wide NAM generated as a by-product of the enzymatic activities of NAD^+^-consuming proteins via two steps. Initially, the NAM phosphoribosyltransferase (NAMPT) recycles NAM into NMN, which is the rate-limiting process of this salvage pathway. The intermediate NMN is then converted into NAD^+^ via the different NMNATs in diverse intracellular compartments.

### NAD^+^ consumption

#### Sirtuins

The discovery that NAD^+^ acts as a cosubstrate for the sirtuin family of deacetylases and thereby effectively coordinates key metabolic processes, stress responses and ageing biology, heralded a new era of NAD^+^ research at the very beginning of the twenty-first century [31–33]. Seven different sirtuins have been identified in mammals, each with distinct subcellular localizations (nucleus for SIRT1 and SIRT6; nucleolus for SIRT7; mitochondria for SIRT3, SIRT4 and SIRT5; and cytosol for SIRT1, SIRT2 and SIRT5), enzymatic activities and physiological functions. All members of the sirtuin family share the evolutionarily conserved NAD^+^-binding domain but differ in their Michaelis constant (*K*_*m*_) values for NAD^+^, which determine differential responses to varying NAD^+^availability and enzymatic activities of deacetylases. For example, NAD^+^ might not necessarily be the rate-limiting factor of enzymatic activities in some sirtuins with *K*_*m*_ values below the physiological range (~ 300–700 µM) of NAD^+^, including SIRT2, SIRT4, SIRT5 and SIRT6. On the contrary, those with *K*_*m*_ values from 94 to 888 µM, such as SIRT1 and SIRT3, are highly dependent on NAD^+^ availability [26, 34, 35]. Therefore, a rise of NAD^+^ levels would dramatically enhance SIRT1 activation during fasting and exercise [36].

Sirtuins use the ADP-ribose produced upon NAD^+^ cleavage as an acetyl acceptor to generate acetyl-ADP-ribose with NAM release. Specifically, sirtuins participate in multiple physiological reactions that regulate cellular metabolism through the NAD^+^-dependent deacetylation of target proteins including histones, such as H1, H3 and H4, transcription factors and co-activators, including p53, NF-κB, peroxisome proliferator-activated receptor-γ co-activator-1α (PGC-1α) and forkhead box O1 (FOXO1), and signalling regulators of metabolism, including protein kinase A, 5′-AMP-activated protein kinase (AMPK) and mechanistic target of rapamycin (mTOR) [37–43]. However, some members of the sirtuin family can catalyze the removal of several other acyl groups (such as malonyl, succinyl and propionyl) [44–47], the physiological functions of which are so far poorly understood.

#### PARPs

The poly(ADP-ribose) polymerase (PARP) protein family has a substantial role in DNA repair and maintenance of genomic stability, in which single or covalently linked polymers of ADP-ribose moieties are added to PARP itself and the nuclear target proteins, which is accompanied by NAD^+^ cleavage into NAM, in a reversible post-transcriptional protein modification process called ‘poly(ADP-ribosyl)ation’ (PARylation) [25, 48]. Among 17 members in humans and 16 members in mice, PARP1 alone is responsible for about 90% of all NAD^+^ used by the PARP protein family [49]. However, since their *K*_*m*_ values for NAD^+^ are far below the physiological range of NAD^+^, PARPs can outcompete sirtuin family proteins for NAD^+^ bioavailability [34]. The strong correlation among PARP1 overactivation, limiting NAD^+^ pools and inhibition of SIRT1 activity is observed in a broad spectrum of human diseases, including xeroderma pigmentosum group A, progeroid diseases, ataxia telangiectasia and Cockayne syndrome. Notably, treatment of mice with Cockayne syndrome with PARP1 inhibitors or NAD^+^ supplementation reversed inactivation of SIRT1 and mitochondrial defects, resulting in slowed ageing, extended lifespan and amelioration of severe phenotypes caused by PARP1 hyperactivation in response to extensive DNA damage and genotoxic stresses [50, 51]. Owing to sharing the same nuclear NAD^+^ pool, the ability of PARP1 to attenuate SIRT1 activity in an NAD^+^-dependent manner is also implicated in some other metabolic disorders. For example, the PARP1 activity was sufficiently robust to diminish SIRT1 activity as a direct result of depletion of NAD^+^ pools in diet-induced obesity. Treatment with PARP inhibitors or deletion of PARP1 in mice fed a high-fat diet increased NAD^+^ levels, improved SIRT1 activity and rescued mitochondrial function, thereby protecting the obese mice from insulin resistance [52–54]. Thus, PARPs and sirtuins in the same subcellular compartmentation have counterbalancing roles in the regulation of NAD^+^ homeostasis [55].

PARP2 has a catalytic structural domain similar to that of RARP1, and is essential for several cellular processes in an NAD^+^-dependent manner, including DNA repair and transcriptional regulation [56–58]. However, in some metabolic disorders, *Parp2*-knockout mice showed enhanced SIRT1 activity and improved metabolic functions, and were protected against high-fat diet-induced obesity, which were not due to augmented NAD^+^ levels, but to an increase in SIRT1 expression, as PARP2 acts as a direct negative regulator of the SIRT1 promoter [59]. Thus, more studies are required to further understand the specific functions of different PARP family members.

#### CD38 and CD157

Another family of NAD^+^ consumers is the cyclic ADP-ribose (cADPR) synthases, which are usually activated during inflammation and hydrolyze a variety of nucleotide metabolites, including ATP and ADP [60]. More specifically, CD38 and its homologue CD157, two of the most prominent cADPR synthases, possess both NAD^+^ glycohydrolase and ADP-ribosyl cyclase activities. In this catalytic reaction, the glycosidic bond within NAD^+^ is hydrolyzed to generate NAM and ADP-ribose, whereas the ADP-ribosyl cyclase activity generates key Ca^2+^-mobilizing second messenger molecules cADPR. In addition to NAD^+^, NMN is emerging as an alternative substrate of CD38 to produce NAM and ribose monophosphate [61, 62]. Of note, CD38 possesses a low *K*_*m*_ value for NAD^+^ in the range of 15–25 µM [63, 64]. Consequently, tissue levels of NAD^+^ in *Cd38*-deficient mice were 10- to 20-fold higher than in wild-type animals [65]. Owing to the lower *K*_*m*_ value, the NADase CD38 has a central role in age-related NAD^+^ decline [66–68]. Consequently, CD38 deficiency elevated SIRT1 and SIRT3 activities by increasing intracellular NAD^+^ levels, thus reestablishing mitochondrial and lysosomal functions to inhibit generation of senescence-associated small extracellular vesicles and acquisition of senescence-associated secretory phenotypes in mice with angiotensin II-induced vascular smooth muscle cell senescence and vascular remodeling [69]. 78c, a highly potent and specific thiazoloquin(az)olin(on)e CD38 inhibitor, restored NAD^+^ levels in aged mice, resulting in increased longevity and enhanced activation of other NAD^+^-consuming enzymes, including sirtuins and PARPs [70].

CD157 degrades NR, an alternative substrate, to generate NAM and ribose, which is upregulated in ageing tissues [71, 72]. However, the physiological significance of CD157 enzymatic functions in ageing remains largely uncharacterized. It is seemingly dispensable in maintaining NAD^+^ homeostasis, because of its poor cADPR-synthesis efficiency that is several hundred fold lower than that of CD38 [73]. Nevertheless, CD157 can serve as a novel biomarker that specifically identifies tissue-resident vascular endothelial stem cells with homeostatic and regenerative properties [74, 75].

#### SARM1

Unlike the ecto-enzyme CD38, SARM1 is an intracellular NAD^+^-consuming glycohydrolase. In normal physiological context, NAD^+^ binds to the armadillo/heat repeat motifs (ARMs), domain of SARM1, thereby facilitating the inhibition of the Toll/interleukin receptor (TIR) domain NADase through the domain interface. After axonal injury, disruption of the NAD^+^-binding site or destruction of the ARM-TIR interaction leads to constitutive activation of SARM1 characterized by the dimerization of the TIR domain, thereby consuming NAD^+^ to generate mostly ADPR and NAM, but also a very small portion of cADPR. As a result, catastrophic NAD^+^ depletion evokes metabolic collapse and axonal degeneration [76–79].

A recent study provided further insight into the underlying mechanisms of SARM1 activation *in vivo.* Upon injury, NMN binding induces a more-closed ARM domain conformation, which leads to destabilization of the peripheral ARM domain ring and disruption of ARM-TIR interaction. This permits self-association of TIR domain to form a functional catalytic site and activate the NADase function, hence triggering axon degeneration [80]. Therefore, SARM1 is a metabolic sensor of the NMN/NAD^+^ ratio in neurons and acts as a potential therapeutic target to prevent or ameliorate neurodegenerative diseases and traumatic brain injuries [81].

### NAD^+^ metabolism in the immune response

NAD^+^, as a cofactor for diverse metabolic reactions and a cosubstrate for several NAD^+^-consuming enzymes, is a mediator of key cellular functions and adaptation to environmental changes [82]. Despite the increased research interest in immunometabolism in the last decade, how NAD^+^ influences immune cell functions and inflammatory responses remains to be fully elucidated. Here, we focus on the recent reports of NAD^+^-mediated immunoregulation of innate and adaptive immunity.

#### Innate immunity

The distinct fluctuations of intracellular NAD^+^ levels are linked to plastic immunological functions of myeloid cells. For example, NAD^+^ availability can influence neutrophil functions. Genetic deletion of optic atrophy 1 (OPA1) in neutrophils, a mitochondrial inner membrane protein, impaired the activity of mitochondrial electron transport complex I and destroyed NAD^+^ regeneration, consequently reducing available NAD^+^ levels, and hence the rate of glycolysis, thereby resulting in decreased ATP production and thus breaking the formation of neutrophil extracellular traps [83]. Diminished NAD^+^ is correlated with mast cell degranulation and anaphylactic responses. Supplementation with the NAD^+^ precursors NMN and NR attenuated IgE-mediated anaphylactic responses in the mouse models of passive systemic anaphylaxis and passive cutaneous anaphylaxis in a SIRT6-dependent manner [84]. In addition, NAD^+^ salvage is engaged to NK cell functions and activation through safeguarding mitochondrial homeostasis and dictating energy metabolism. Boosting NAD^+^ metabolism through NMN supplementation can enhance the antitumor immunity of NK cells, attributed to increased cytokine production and augmented cytotoxic activity [85].

**Fig. 2:**
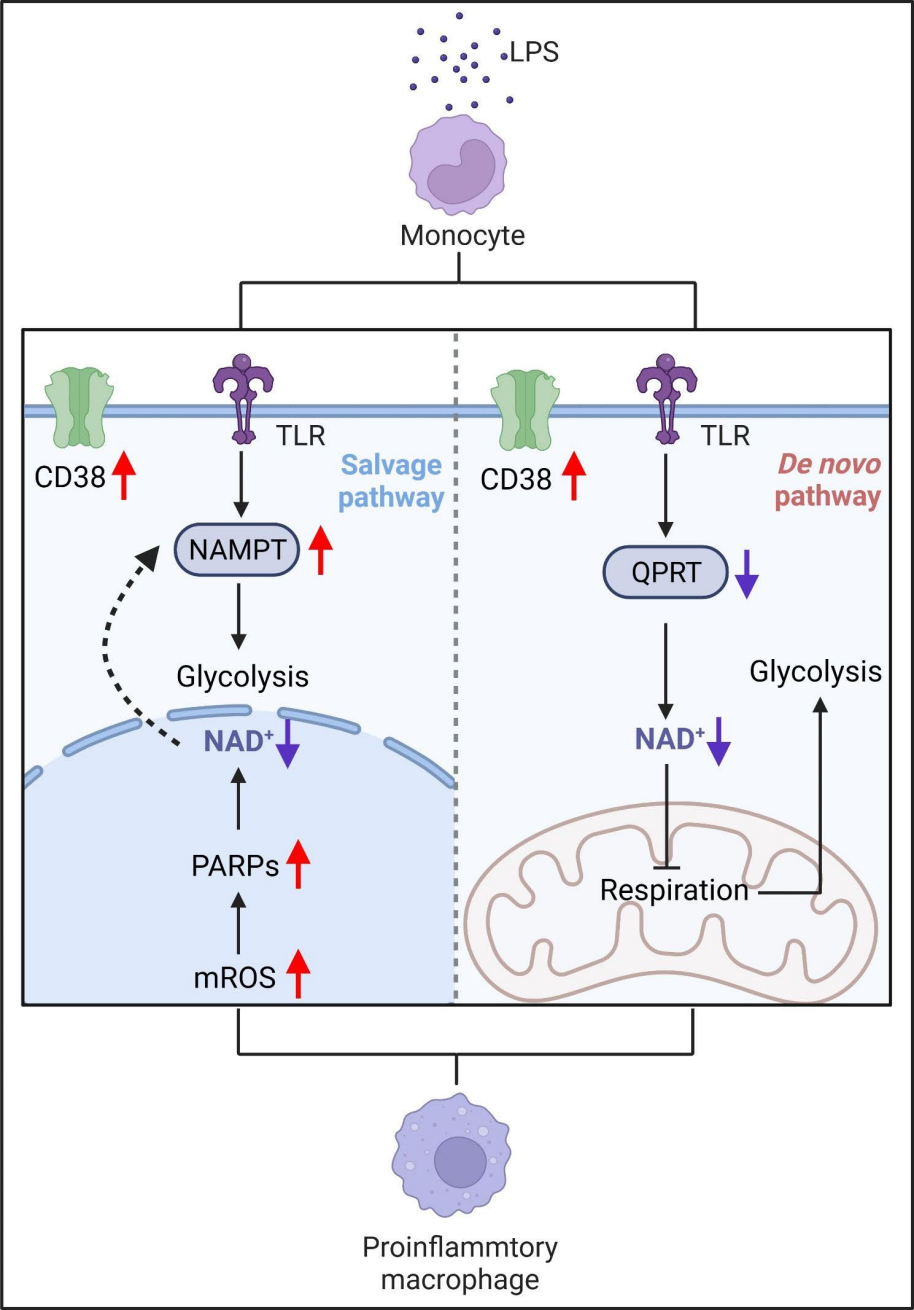
NAD^+^ regulates macrophage polarization. LPS challenge induces the generation of mitochondrial reactive oxygen species (mROS). This increase in ROS results in DNA damage and subsequent PARP activation, which consume NAD^+^ to repair damaged DNA and maintain genomic integrity. The NAD^+^ salvage pathway replenishes the NAD^+^ pools through increased NAMPT expression, thus initiating glycolytic reaction and programming pro-inflammatory macrophage polarization. In another context, diminished NAD^+^ levels are attributed to decreased QPRT expression in the *de novo* synthesis pathway. Low NAD^+^ concentration impairs mitochondrial respiration, thus inversely increasing glycolysis and facilitating inflammatory responses of macrophages. In addition, CD38 acts as another important NAD^+^-consuming enzyme during pro-inflammatory macrophage polarization.

Additional lines of evidence support that intracellular NAD^+^ acts as a critical regulator of macrophage functions and phenotypic polarization (Fig. 2). Some of the earliest work demonstrated that depletion of NAD^+^ pools in macrophages through inhibition of NAMPT decreased the secretion of pro-inflammatory cytokines, such as TNF-α, and caused morphological changes, such as reduced spreading [86, 87]. Consistent with these results, a recent study showed that the NAMPT-mediated NAD^+^ salvage pathway is required for the pro-inflammatory (M1) macrophage polarization in response to LPS [88]. In this context, activation-induced mitochondrial reactive oxygen species (mROS) production caused a rapid, extensive DNA damage response and consequently PARP1 activation with associated NAD^+^ consumption. As a result, a rapid decrease in NAD^+^ levels triggered increased expression of NAMPT to maintain the NAD^+^ content, thereby sustaining glycolysis in these pro-inflammatory macrophages. Pharmacological inhibition of NAMPT by FK866 blocked glycolytic shifts due to loss of function of NAD^+^-dependent GAPDH, leading to an impaired production of a subset of inflammatory mediators *in vitro* and a reduced systemic inflammation *in vivo* in response to sepsis. In contrast, supplementation with NAD^+^ precursor NMN restored the impaired metabolic effects caused by FK866 treatment [88, 89]. Therefore, blocking the NAMPT-dependent NAD^+^ biosynthetic pathway is emerging as an attractive novel therapeutic strategy in acute intestinal inflammation. FK866-induced NAD^+^ depletion skewed monocytes/macrophages away from a pro-inflammatory towards an anti-inflammatory phenotype, thereby effectively ameliorating experimental colitis *in vivo* and diminishing pro-inflammatory cytokine release from human inflammatory bowel disease (IBD)-derived lamina propria mononuclear cells comparably to dexamethasone or infliximab *in vitro* [90]. NAMPT-mediated glycolytic processes are also required for antitumor immunity of tumor-infiltrating macrophages. In this context, STAT1 binds to a conserved element within the first intron of NAMPT, termed NAMPT-Regulatory Element-1 (NRE1), and enhances its expression, thus driving aerobic glycolysis and initiating expressions of a subset of inflammatory genes in the tumor-associated macrophages stimulated with IFN-γ [91]. Moreover, the hyperactivation of PARP1 in response to ROS-induced DNA damage, fueled by NAMPT-derived NAD^+^, could also lead to parthanatos cell death, thereby promoting neutrophil infiltration and thus mounting skin inflammation [92]. However, other recent studies showed some conflicting results, in which DNA damage or PARP1 activation was not detected during M1 macrophage polarization. By contrast, enhanced expression of CD38 primarily contributed to the decrease of NAD^+^ levels [62, 93, 94]. These results were consistent with the reports showing that M1 macrophages were protected from ROS-induced DNA damage as a result of increased expression of multiple antioxidant genes, such as superoxide dismutase 2 (SOD2) [95, 96].

However, the NAD^+^ functions in innate immune cells are complex and context-dependent. For example, boosting NAD^+^ through NR administration could confer anti-inflammatory effects on tissue macrophages, in which NR supplementation attenuated the type I IFN signaling pathway, an important myeloid regulatory program, through the increased inosine level and the subsequent suppression of autophagy [97]. In inflammatory and ageing macrophages, the *de novo* NAD^+^ synthesis pathway functions to maintain NAD^+^ homeostasis and to restrict inflammation. Depletion of NAD^+^ due to genetic ablation (in *Ido1*-knockout and *Qprt*-knockout mice) or pharmacological inhibition (using 1-methyl-L-tryptophan and phthalic acid) of this pathway suppressed mitochondrial NAD^+^-dependent signaling and respiration, increased glycolysis and impaired resolution of inflammation. These macrophages possessed typical pro-inflammatory signatures, such as increased expression of the pro-inflammatory markers CD86 and CD64, decreased expression of the anti-inflammatory markers CD206 and CD23, and impaired phagocytosis. Supplementation with NMN or overexpression of QPRT to refill the NAD^+^ pools restored mitochondrial respiration and SIRT3 activity and normalized TCA cycle intermediates to homeostatic, anti-inflammatory levels during LPS-induced M1 macrophage polarization [30]. Why did the same NAD^+^-boosting strategy through the addition of NMN result in opposite results? A potential explanation is that the maintenance of NAD^+^ pools may help to restore the activities of different NAD^+^-dependent proteins to drive distinct metabolic processes, respectively enhancing GAPDH-mediated glycolytic reaction and elevating SIRT3-induced OXPHOS in macrophages activated with LPS at different time points. A recent report suggested that the preference of NAD^+^ biosynthetic pathways could be shifted in highly plastic macrophages at different phases of disease progression. While low dose endotoxin triggered NAMPT-dependent NAD^+^ salvage pathway to support pro-inflammatory response, high dose endotoxin drove a switch from NAD^+^ salvage to IDO1-dependent NAD^+^*de novo* biosynthesis at late stage, leading to persistent anti-inflammatory properties and immune tolerance [98]. In addition, NAMPT-dependent NAD^+^ biosynthetic pathway not only alters macrophage polarization states but also dictates macrophage phagocytosis [99]. In contrast to the aforementioned alleviation of experimental colitis by NAMPT inhibition, mice with NAMPT deletion in macrophages had more pronounced colitis with lower survival rates. In this context, insufficient NAD^+^ abundance caused reduced NADPH levels and defective oxidative burst, thereby impairing the phagocytosis functions of inflammatory macrophages and contributing to the accumulation of apoptotic corpses within the mucosal layer [99]. Collectively, these findings indicated that the role of NAD^+^ in macrophage biology may be context and signaling pathway specific.

To summarize, the reported contradictory roles of NAD^+^ likely reflect the complexity and intricacy of the NAD^+^-mediated immunomodulation of innate immunity. Further studies are necessary to clearly delineate the major NAD^+^ biosynthetic pathways and NAD^+^ consumption routes during macrophage phenotypic transition to determine whether the observed decline in NAD^+^ levels depends on context and time and to determine the molecular mechanisms through which NAD^+^ levels alter macrophage polarization states and functions.

#### Adaptive immunity

The studies of biological effects of NAD^+^ on adaptive immune responses started in the early 2000s, prior to those on macrophages. Early work suggested that extracellular NAD^+^ can induce cell death in specific T cell subpopulations, such as naïve T cells and regulatory T cells (Tregs), representing the prototype of a new category of danger signals and being known as NAD^+^-induced cell death (NICD) [100–102]. Mechanistically, ADP-ribosyl-transferase 2 (ART2) transfers the ADP-ribose moiety from NAD^+^ to the neighbouring cell surface purinergic P2 × 7 receptor (P2 × 7R), thereby initiating the apoptotic programme, characterized by deregulated calcium flux, pore formation, phosphatidylserine exposure, shedding of CD62L and cell shrinkage, and hence inducing cell death in peripheral T cells exposed to NAD^+^ [103]. This mechanism is involved in the immune resistance of non-small cell lung cancer. Indeed, ART1 overexpression on lung cancer cells can enhance susceptibility to ART1-mediated ADP-ribosylation and to NICD in tumor-infiltrating P2 × 7R^+^CD8^+^ T cells, thereby constraining effective antitumor immunity and contributing to acquired resistance to immunotherapy [104]. Apart from ART, CD38 on the cell surface is another important regulator of NICD. They play counterbalancing roles in the regulation of extracellular NAD^+^ metabolism. *Cd38*-knockout mice showed an exacerbated NICD in peripheral T cells upon intravenous injection of NAD^+^, suggesting that CD38 is an important survival factor during inflammation, in which it attenuates ART pro-apoptosis activity by reducing local NAD^+^ bioavailability [105].

Different T cell subpopulations possess distinct sensitivities to NICD. As a result, ART2-mediated death programme contributes to the dynamic regulation of T cell homeostasis. Extracellular NAD^+^ preferentially induces apoptosis of resting T cells and Tregs, while thymocytes and primed T cells deficient in ART2 expression are resistant to NICD [102, 105–107]. Moreover, systemic administration of NAD^+^ could reduce the frequency of tumor-infiltrating Tregs and augment antitumor immunity through ART2-P2 × 7 pathway-initiated NICD in several tumor models [108]. Given that Tregs hinder effective antitumor immunity in humans, Treg depletion has been emerging as a potential therapeutic strategy to evoke antitumor immune responses [109].

Additionally, NAD^+^ metabolism is essential for T cell activation and differentiation [110–112]. Manipulation of intracellular NAD^+^ levels via supplementation with NAD^+^ precursors and altering NAD^+^-biosynthetic or-consuming pathways can release immunomodulatory properties in various T cell-mediated inflammatory disorders. NAD^+^ depletion by NAMPT inhibitor FK866 decreased proliferative capacities and the production of inflammatory cytokines in activated T cells, and hence alleviated demyelination and disability in experimental autoimmune encephalomyelitis (EAE) [113]. Furthermore, local targeting of NAD^+^ salvage pathway by microparticle-mediated intratumoral delivery of NAMPT inhibitor GMX1778 induced striking immunologic changes in the tumor microenvironment of murine glioblastoma, characterized by upregulation of immune checkpoint PD-L1, recruitment of cytotoxic T cells, and reduction of tumor-associated macrophages, and potentiated checkpoint immunotherapy [114]. On the contrary, administration of different NAD^+^ precursors to boost intracellular NAD^+^ levels increased Ca^2+^ mobilization, promoted cell proliferation and IL-2 release in response to mitogens, thereby upregulating activated T cell functions [115]. On the other hand, the modulation of mitochondrial NAD^+^/NADH levels can impact lysosome functions during inflammatory T cell responses. Conditional deletion of the mitochondrial transcription factor A (Tfam) in CD4^+^ T cells led to an imbalance in the NAD^+^/NADH ratio of mitochondria, which induced metabolic reprogramming toward glycolysis and thereby contributed to their pro-inflammatory phenotype, consequently exacerbating the *in vivo* immune response. Increasing intracellular NAD^+^ contents by the NAD^+^ precursor NAM improved lysosomal functions and dampened pathogenic Th1 responses in these respiration-impaired cells [116]. Furthermore, *Tfam*-specific knockout in mouse T cells disrupted mitochondrial genome integrity and induced an aberrant Th1-type pro-inflammatory response, characterized by enhanced expression of T-bet, IFN-γ and TNF-α, resulting in increased senescence, neuromuscular and vascular dysfunction, and reinforced molecular features that recapitulate premature ageing. Interestingly, boosting levels of NAD^+^ through NR treatment alleviated these phenotypes by reducing senescence and systemic inflammation [117–119]. Thus, these studies suggest that NAD^+^ is a critical metabolite for T cell activation and differentiation, and its metabolism mediates distinct biological processes and functions of T cells.

Besides NAD^+^ biosynthetic pathways, targeting NAD^+^ degradation enzymes may provide a viable therapeutic avenue to treat cancer [67, 120]. CD38 overexpression was associated with the generation of dysfunctional and exhausted T cells in those cancers with acquired resistance to immune checkpoint therapy. Co-inhibition of PD-L1 and CD38 produced a favorable antitumor microenvironment and expanded the efficacy of immune checkpoint inhibitors [121, 122]. Moreover, boosting intracellular NAD^+^ levels using anti-CD38 antibody enhanced antitumor properties of hybrid Th1/17 cells through the upregulation of NAD^+^-SIRT1-FOXO1 axis [123, 124]. On the other hand, the NAD^+^-consuming enzyme CD38 responsible for the decline in NAD^+^ levels impaired cytotoxic CD8^+^ T cell responses and increased propensity to infections in patients with systemic lupus erythematosus (SLE) [125]. Mechanistically, low NAD^+^ levels due to CD38 activation limited NAD^+^ bioavailability for SIRT1 deacetylase activity, thereby enhancing acetylated EZH2 and repressing downstream RUNX3 expression, which resulted in decreased levels of cytotoxic-related molecules and increased risk for infections [126]. Furthermore, persistent activation of the TCR and the type I IFN-α signalling triggered mitochondrial changes via increasing NAD^+^ consumption due to both CD38 overexpression and PARP activation in CD8^+^ T cells, resulting in lower spare respiratory capacity, decreased bioenergetic fitness and thus increased tendency to die. NAD^+^ supplementation with NMN restored the NAD^+^ pools, increased the mitochondrial respiration and improved T cell viability upon TCR restimulation in SLE [127, 128].

Similarly, the NAD^+^-dependent deacetylase activity of SIRT1 is also implicated as a pathogenic factor attributing to its pro-inflammatory properties in multiple autoimmune diseases such as multiple sclerosis, in which deacetylation of RORγt increases its transcriptional activity, thereby enhancing Th17 cell generation and shaping pathological phenotypes. Therefore, genetic ablation or pharmacological inhibition of SIRT1 in Th17 provided a protective benefit in the EAE model [129, 130]. More importantly, SIRT1 seems to possess anti-inflammatory functions in the memory population of highly cytotoxic CD8^+^CD28^−^ T cells during immunosenescence [131]. In this context, the decreased SIRT1 and FOXO1 levels led to metabolic reprogramming of CD8^+^CD28^−^ T cells, characterized by an enhanced glycolytic capacity and a high secretion of cytotoxic granzyme B [132]. Additionally, the NAD^+^-dependent deacetylase SIRT2 can also govern the metabolic fitness of immune cells by blocking the activities of key enzymes in multiple metabolic pathways, thus impairing effector functions of tumor-reactive T cells in the tumor microenvironment. Pharmacologic inhibition of SIRT2 enhanced antitumor immunity by promoting metabolic reprogramming toward a profound hyper-metabolic state with enhanced capacity for aerobic glycolysis in tumor-infiltrating T cells [133].

**Fig. 3:**
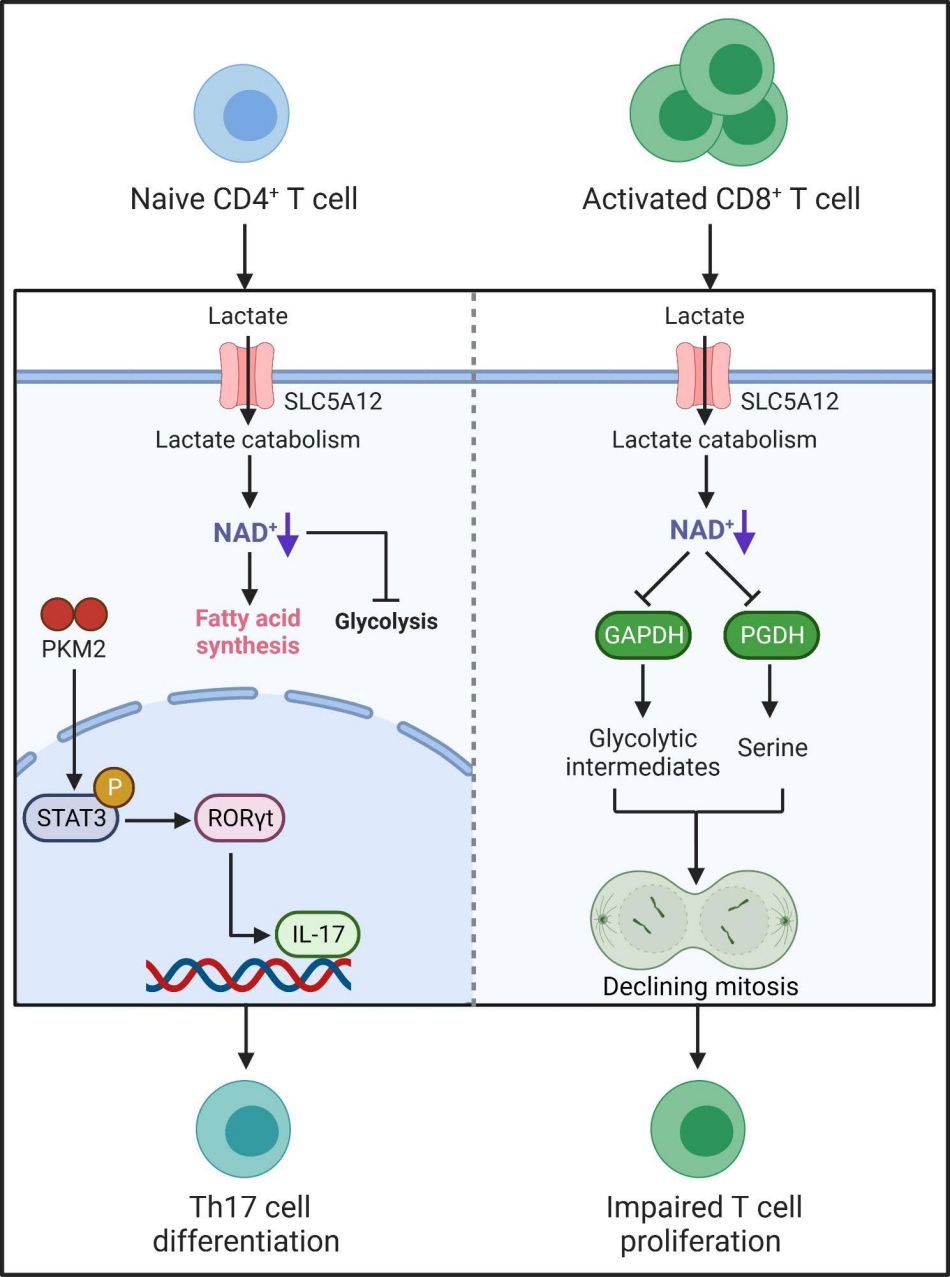
NAD^+^ regulates T cell fates through environmental lactate. SLC5A12-mediated lactate uptake into CD4^+^ T cells in the inflamed tissue reshapes their effector phenotypes, resulting in RORγt activation and subsequent IL-17 transcription via nuclear PKM2/STAT3 and enhanced fatty acid synthesis. It also leads to these renascent Th17 cell retention in the inflamed tissue as a result of reduced glycolysis and enhanced fatty acid synthesis. On the other hand, the continuous lactate catabolism through lactate dehydrogenase consumes amounts of NAD^+^ contents. The insufficient NAD^+^ pools cannot sustain NAD^+^-dependent enzymatic reactions involving glyceraldehyde 3-phosphate dehydrogenase (GAPDH) and 3-phosphoglycerate dehydrogenase (PGDH). The dysfunction of GAPDH and PGDH leads to the depletion of post-GAPDH glycolytic intermediates and the 3-phosphoglycerate derivative serine that are important fuels for T cell proliferation. The environmental lactate eventually suppresses effector T cell proliferation.

Cellular NAD^+^ availability determines whether cells engage in aerobic glycolysis [134]. Upon activation, T cells carry out glycolysis to meet the high demand for energy, which transforms glucose fuel into ATP [135]. However, increased lactate in inflamed tissues could be a key point of T cell suppression, in which environmental lactate is metabolized to pyruvate, accompanied by NAD^+^ reduction to NADH (Fig. 3). Limiting NAD^+^ supply compromises NAD^+^-dependent enzymatic reactions involving GAPDH and 3-phosphoglycerate dehydrogenase (PGDH), resulting in the depletion of post-GAPDH glycolytic intermediates as well as the 3-phosphoglycerate derivative serine and thus suppressing T cell proliferation [136]. In contrast, pathological NAD^+^ metabolism mediated by hyperactivated NAMPT promotes aerobic glycolysis and mitochondrial respiration in CD4^+^ T cells, thereby inducing their excessive IFN-γ production and thus driving lupus nephritis (LN) progression, suggesting that NAMPT drives the pathogenicity of CD4^+^ T cells in LN [137]. Lactate-induced metabolic reprogramming was characterized by not only reduced glycolysis but also enhanced *de novo* fatty acid synthesis in CD4^+^ T cells with a drop in the NAD^+^/NADH ratio through the upregulation of the lactate transporter SLC5A12, which reshaped their effector phenotypes, resulting in increased IL17 production via an increase of PKM2 nuclear translocation with concomitant enhanced STAT3 phosphorylation, and induced tissue retention of these pathogenic T cells in rheumatoid arthritis [138, 139]. Taken together, these studies highlight the importance of NAD^+^/NADH ratio in the modulation of metabolic processes and subsequent T cell functions.

Overall, as inflammation is a very complex and multipurpose process, further delineation of how the fluctuations of intracellular NAD^+^ pools influence different inflammatory states will aid a better understanding of the principle of immunoregulation by NAD^+^ metabolism.

### The NAD^+^ therapeutics in human inflammatory diseases

Because of its crucial roles in the regulation of immune responses, NAD^+^ metabolism dictates the development of inflammatory diseases. For this reason, the therapeutic and preventive potential of targeting NAD^+^ has been widely explored in a variety of preclinical models and disease settings, including IBD, EAE and SLE. The promising outcomes of these studies have prompted a series of clinical trials of NAD^+^ therapeutics for their safety and efficacy in inflammatory conditions. Below, we summarize recent progresses of these key clinical trials.

#### Muscle

NR supplementation could improve a broad spectrum of skeletal muscle physiological functions and alleviate pathological processes in rodent disease models [140–142]. More importantly, participants receiving 1 g oral NR per day for 21 days in a placebo-controlled, randomized, double-blind, crossover trial showed augmented skeletal muscle NAD^+^ metabolome and decreased levels of circulating inflammatory cytokines, without apparently altering skeletal muscle mitochondrial bioenergetics [143]. Thus, the NAD^+^ precursor NR, as a nutritional supplement, is bioavailable to human skeletal muscle and has prominent immunomodulatory properties under inflammatory conditions. Dysregulation of inflammation in skeletal muscle influences whole-body glucose homeostasis and insulin sensitivity [144–146]. Unfortunately, in a recent randomized and double-blind study, while NR could effectively increase NAD^+^ levels, patients with overweight and obesity showed no change in beneficial energy metabolism and amelioration of insulin resistance [147]. Therefore, whether the manipulation of NAD^+^ metabolism with NAD^+^ precursors could treat metabolic diseases through their anti-inflammatory abilities remains to be determined.

#### Heart

A decrease in cardiac NAD^+^ levels is causally linked to metabolic abnormalities and mitochondrial dysfunction, which contribute to the pathologies of heart failure [148–150]. Different strategies for restoring cardiac NAD^+^ contents have yielded promising results in animal models of cardiomyopathy [151–155]. However, most of these studies focused on the beneficial effects of NAD^+^ boosting on the activity of NAD^+^-dependent deacetylases in the myocardium, in which augmenting NAD^+^ levels improved myocardial mitochondrial function and energy metabolism. On the other hand, chronic sterile inflammation, characterized by aberrant activation of the immune system, enhanced expression of pro-inflammatory cytokines, such as TNF-α, IL-1, IL-6 and IL-18, and activation of immune complexes, such as the NLRP3 inflammasome, is now being recognized as a key driver of disease progression in heart failure [156, 157]. The immunosuppressive efficacy of NAD^+^ boosters has not been extensively evaluated in the heart failure-related clinical trials. Fortunately, a recent study in humans provided promising preliminary results. Oral NR (2000 mg daily) administration for 5–9 days in four patients with stage D heart failure resulted in an increase of whole blood NAD^+^ levels, a consistent enhancement of basal and maximal respiration in peripheral blood mononuclear cells, and an up to 30-fold reduction of NLRP3 and inflammatory cytokines. The reduction of systemic inflammation through inhibition of pro-inflammatory activation of circulating immune cells allows the cardiovascular system to break from the vicious cycle that perpetuates the disease, thereby protecting against cardiomyopathy and thus contributing to better clinical outcomes [158]. Nevertheless, due to the small case number and short treatment period of this trial, solid conclusions regarding the efficacy and safety of NR remain to be drawn. Future large-scale and long-term clinical trials to investigate potential anti-inflammatory effects of NAD^+^ precursors are imperative to the development of targeted novel therapeutics for heart failure.

#### COVID-19

Metabolic abnormalities and hyperinflammatory responses are associated with a higher risk of mortality and more severe forms of COVID-19, in which the deficiencies in NAD^+^ and glutathione metabolism may be primary causes [159–161]. Boosting NAD^+^ and GSH levels is emerging as a viable therapeutic approach to treating COVID-19 [162, 163]. A recent study in humans also demonstrated the enormous therapeutic potential of this strategy. Patients treated with a mixture of combined metabolic activators (CMAs) consisting of glutathione and NAD^+^ precursors, in placebo-controlled, open-label phase 2 study and double-blinded phase 3 clinical trials, showed shorter time to complete symptom-free recovery and improved plasma biochemical parameters associated with inflammation and antioxidant metabolism [164]. Thus, the immunomodulatory ability of NR can help to alleviate disease progression of COVID-19 patients and reduce disease severity. In addition, targeting NAD^+^ degradation enzymes and pathways to raise NAD^+^ levels may represent new therapeutic approaches to treating COVID-19. Emerging evidence supports that the CD38 ectoenzyme and products controlled by the CD38/NAD^+^ axis may favor the onset of lung immunopathology. With a view to the various effects of CD38 on the functions of immune cells, the use of CD38-targeted therapies may be a viable treatment option for those life-threatening COVID-19 patients [165, 166].

## Conclusions and future perspective

After over 110 years since its initial discovery, NAD^+^ is emerging as a central metabolite in life science field, and altered NAD^+^ homeostasis is becoming an established common feature of multiple pathologic diseases. With the use of stable-isotope tracing technology and development of NAD^+^ biosensors, our understanding of how NAD^+^ influences immune cell functions and inflammatory disease progression is rapidly evolving. The changes of NAD^+^ biosynthetic and degradative pathways induce rapid fluctuations of intracellular NAD^+^ pools, which regulate the activity of NAD^+^-dependent enzymes and dictate metabolic reactions, subsequently altering functionality attributes of innate and adaptive immune cells and contributing to inflammatory conditions. Most importantly, the immunoregulatory function of NAD^+^ is highly plastic. According to functional differences of NAD^+^-dependent enzymes, NAD^+^ can either suppress or enhance immune responses. Therefore, learning how to control the plasticity of immunomodulation by NAD^+^ may provide an important new modality for better therapeutic application of NAD^+^ biology, as well as further understanding of the role of NAD^+^ homeostasis in different types of inflammatory diseases.

Most of the preclinical studies in rodents suggest a strong translational potential of targeting NAD^+^ therapies. However, the subsequent parallel human clinical trials have not consistently reproduced similar beneficial results. The translatability of targeting NAD^+^ therapies to humans remains the key question. To date, early-phase clinical trials of short-term NR administration have proven NAD^+^ augmentation to be safe and effective to increase NAD^+^ levels, reduce immune responses and alleviate inflammatory conditions in specific contexts. It is still unknown whether long-term supplementation with NAD^+^ precursors has any side effects. Moreover, targeting NAD^+^ degradation enzymes and pathways, such as CD38 and/or PARP1 inhibitors, should be taken into consideration in the development of inflammatory disease therapeutics. Enhancing NAD^+^ bioavailability through synthetic biomaterials such as nanoparticles is emerging as an important new modality for better therapeutic application in disease settings [167]. Hopefully, the upcoming results of current preclinical and clinical studies will shed some light on our unsolved questions and set the basis for future directions in deciphering the role of NAD^+^ immunometabolism in humans.

## Data Availability

Not applicable.
